# The *peacefulness* gene promotes aggression in *Drosophila*

**DOI:** 10.1186/s13041-018-0417-0

**Published:** 2019-01-03

**Authors:** Mahmoudreza Ramin, Yueyang Li, Wen-Tzu Chang, Hunter Shaw, Yong Rao

**Affiliations:** 1 Department of Neurology and Neurosurgery, McGill Centre for Research in Neuroscience, 1650 Cedar Avenue, Montreal, Quebec, H3G 1A4 Canada; 2 Integrated Program in Neuroscience, 1650 Cedar Avenue, Montreal, Quebec, H3G 1A4 Canada; 3 Department of Medicine, McGill University Health Centre, 1650 Cedar Avenue, Montreal, Quebec, H3G 1A4 Canada; 4 Centre for Research in Neuroscience, McGill University Health Centre, Room L7-136, 1650 Cedar Avenue, Montreal, Quebec, H3G 1A4 Canada

## Abstract

Natural aggressiveness is commonly observed in all animal species, and is displayed frequently when animals compete for food, territory and mating. Aggression is an innate behaviour, and is influenced by both environmental and genetic factors. However, the genetics of aggression remains largely unclear. In this study, we identify the *peacefulness* (*pfs*) gene as a novel player in the control of male-male aggression in *Drosophila*. Mutations in *pfs* decreased intermale aggressiveness, but did not affect locomotor activity, olfactory avoidance response and sexual behaviours. *pfs* encodes for the evolutionarily conserved molybdenum cofactor (MoCo) synthesis 1 protein (Mocs1), which catalyzes the first step in the MoCo biosynthesis pathway. Neuronal-specific knockdown of *pfs* decreased aggressiveness. By contrast, overexpression of *pfs* greatly increased aggressiveness. Knocking down Cinnamon (Cin) catalyzing the final step in the MoCo synthesis pathway, caused a *pfs*-like aggression phenotype. In humans, inhibition of MoCo-dependent enzymes displays anti-aggressive effects. Thus, the control of aggression by Pfs-dependent MoCo pathways may be conserved throughout evolution.

**Electronic supplementary material:**

The online version of this article (10.1186/s13041-018-0417-0) contains supplementary material, which is available to authorized users.

## Introduction

All animal species display aggression, an innate behaviour that is evolutionarily conserved. While natural aggressiveness is important for survival and reproduction, abnormal aggressiveness can cause the waste of energy, severe injuries, wars and destruction. Accumulated evidence supports that aggression is influenced by both environmental and genetic factors [1–3]. For instance, social experience has been shown to play an important role in modulating the levels of aggressiveness in humans as well as animal models [3–6]. Recent studies also begin to reveal genetic factors underlying heritable differences in aggressiveness [7–9].

*Drosophila melanogaster* is an excellent model system for studying neural and genetic basis of aggression. Aggressive behaviours in *Drosophila* were firstly reported by Alfred Sturtevant [10], and later studied in greater details by the groups of Jacobs [11], Hoffmann [12] and Kravitz [13]. Like that in mammals [14], manipulating the levels of neurotransmitters such as serotonin, dopamine and octopamine modulates aggressiveness in *Drosophila* [15–18]. Quantitative-trait linkage analyses by Mackay and coworkers suggest that a number of candidate genes may be associated with aggressive behaviours in *Drosophila*, many of which have homologs in mammals [19]. A recent study also shows that the fly homolog of the gene encoding for neuropeptide Tachykinin/Substance P associated with aggressive behaviors in mammals [20], is also required for aggression in *Drosophila* [21]. These studies support the evolutionarily conservation of certain genetic mechanisms underlying the control of aggression.

In a search for genetic factors involved in the control of fly aggression, we identify the *peacefulness (pfs)* gene as a novel and important player required for male-male aggression. *Pfs* encodes for molybdenum cofactor (MoCo) synthesis 1 protein, an evolutionarily conserved enzyme that catalyzes the first step in the MoCo biosynthesis pathway [22]. MoCo is absolutely required for the activity of molybdoenzymes such as sulphite oxidase, xanthine oxidase and aldehyde oxidase [23]. Interestingly, inhibition of MoCo-dependent xanthine oxidase has been shown to display anti-aggressive effects in humans [24–27].

In this report, we describe our study on the identification and characterization of *pfs* in the control of fly aggression. By taking a combination of behavioural analyses, transgene rescue, cell-type-specific knockdown and overexpression, we investigate the requirements and functions of Pfs in regulating fly aggressiveness.

## Results

### P-element insertion *d03517* decreased intermale aggressiveness

**Fig. 1:**
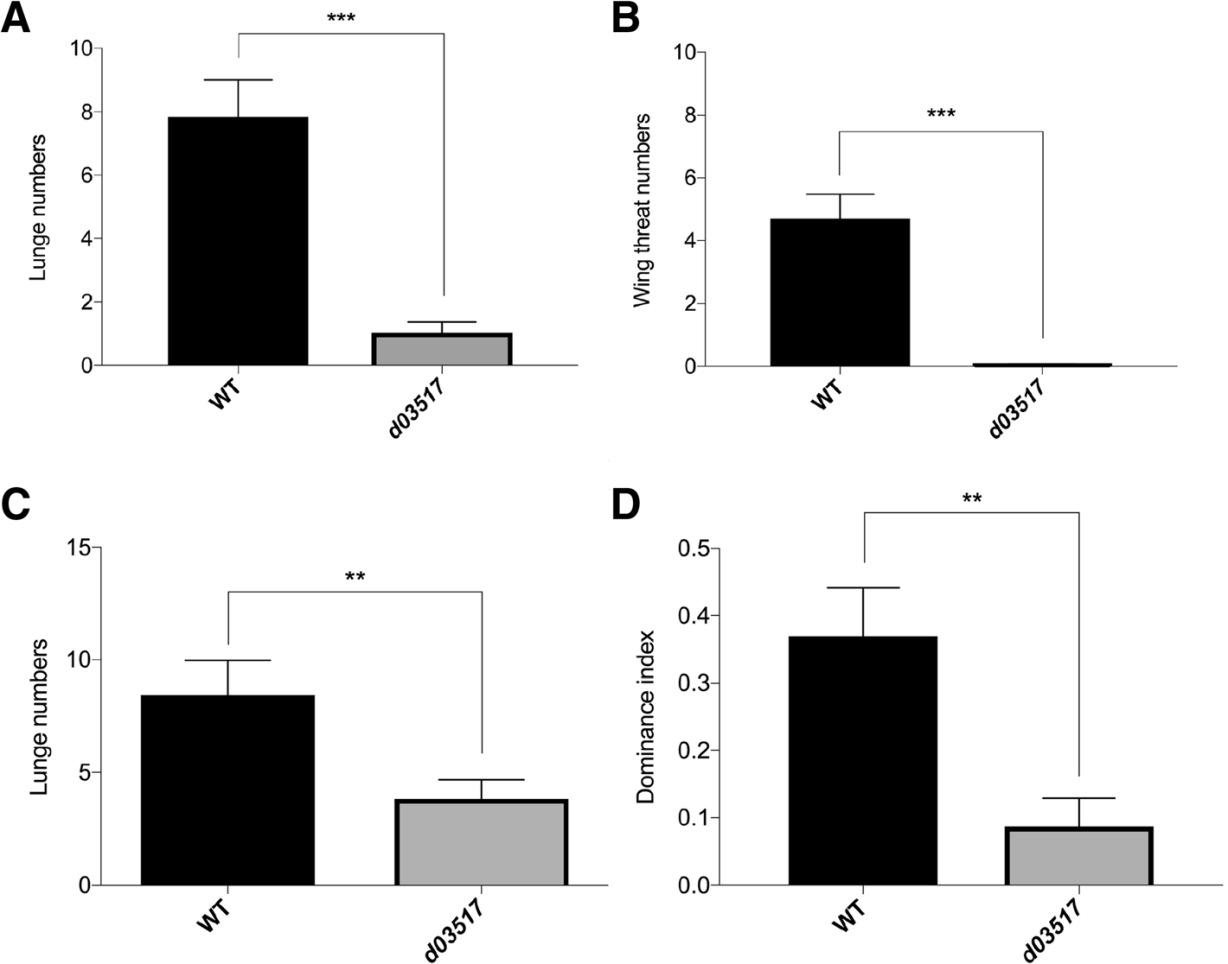
The P-element insertion *d03517* decreased male-male aggressiveness. **a** and **b** Intermale aggressive behaviors for 15-min period in wild-type and *d03517* mutant flies. Prior to behavioral assays, *d03517* flies were backcrossed with *CS* wild-type flies for four generations and thus were in *w*^+^ background. **a** Number of lunges. **b** Number of wing threats. Pairs of flies tested: *wt*, *n* = 27; *d03517/ d03517*, *n* = 27. Mann-Whitney U test, ^***^P < 0.001. **c** Aggressive behaviours for 15-min period were examined when a wild-type male fly was paired with a *d03517* mutant male fly. Pairs of flies tested: *n* = 46. Mann-Whitney U test, ^**^P < 0.01. **d** Dominance index for successful occupancy of food patch by a wild-type or a *d03517* mutant male fly after 10-min period. Pairs of flies tested: *n* = 46. Student’s t-test, **P < 0.01. Error bars represent SEM

In a search for novel genetic factors involved in the control of aggression, we found that mutants homozygous for P-element insertion *P{XP}d03517* (*d03517*) showed a significant decrease in the levels of intermale aggressiveness (Fig. 1a and b). In each experiment, two male flies of same age (isolated for 5–7 days after eclosion) with similar size were introduced into a small chamber, and their behaviours were immediately videotaped for 15-min period. The movies were subsequently analyzed with an automated analysis system CADABRA [28]. Compared to Canton-S (CS) wild-type flies, *d03517* mutant flies displayed much fewer lunges and wing threats (Fig. 1a and b, Additional file 1: Movie 1B). We did not observe tussle, a rare and more intense fighting behaviour, in wild-type (*n* = 27 pairs) or *d03517* mutant flies (*n* = 27 pairs) within 15-min period.

Above phenotypes raise at least two possibilities. For instance, *d03517* mutant flies were incapable of initiating aggression. Alternatively or additionally, they may be incapable of evoking aggression by other flies. To distinguish among these possibilities, we paired a *d03517* mutant male fly with a CS wild-type male fly and examined their behaviours. We found that wild-type flies displayed much higher levels of aggressiveness than *d03517* mutants (Fig. 1c). This result suggests that *d03517* insertion interferes with the internal state required for aggression, but does not affect the ability to evoke aggression by wild-type flies (Fig. 1c).

We then tested if higher aggressiveness in wild-type flies gives them competitive advantage over *d03517* mutants to defending their territory. In such experiments, a wild-type male fly and a *d03517* mutant male fly were placed into a small chamber and allowed to compete for food patch in the center. The frequency for successful occupancy of the food patch was quantitated (see Materials and methods). We found that wild-type flies were much more successful in occupying and defending the food patch than *d03517* mutant flies (Fig. 1d. Additional file 2: Movie 2).

In summary, *d03517* insertion caused a significant decrease in male fly aggressiveness, which may at least partially account for their disadvantage in defending territory when paired with wild-type male flies.

### *d03517* insertion did not affect locomotor activity

To test if the decrease in aggressiveness in *d03517* mutants was due to some general defects in physical capabilities, we examined fly locomotion over 15-min period. A wild-type male fly was paired with a *d03517* mutant male fly, and their movements were videotaped and analyzed. No significant difference in the patterns or total distance of movements was observed between wild-type and *d03517* mutant male flies (Additional file 3: Figure S1A).

We also performed climbing test to examine potential effects of *d03517* insertion on fly motor functions. No significant difference in climbing ability was observed between wild-type and *d03517* mutant male flies (Additional file 3: Figure S1B). These results argue against that the observed decrease in aggressiveness was caused by defective physical capabilities.

### *d03517* insertion did not affect olfactory avoidance response

Olfactory sensation plays important roles in regulating fly behaviours, such as aggression and courtship [29–31]. This raises the possibility that the decrease in aggressiveness of *d03517* mutants was caused by defective olfactory sensation. To test this, we performed experiments to examine the response of wild-type and *d03517* mutant flies to benzaldehyde, a strong odorant repellent (Additional file 4: Figure S2A). We found that like wild-type flies, *d03517* mutant flies could effectively detect and avoid the area with benzaldehyde (Additional file 4: Figure S2B).

### *d03517* insertion did not affect sexual behaviours

When encountering other flies, a male fly has to make certain mutually exclusive decisions, such as fighting or courtship. The observed decrease in intermale aggression of *d03517* mutant male flies may reflect a specific failure of initiating and/or executing fighting, or reflect a switch in decision making due to altered sexual orientation.

**Fig. 2:**
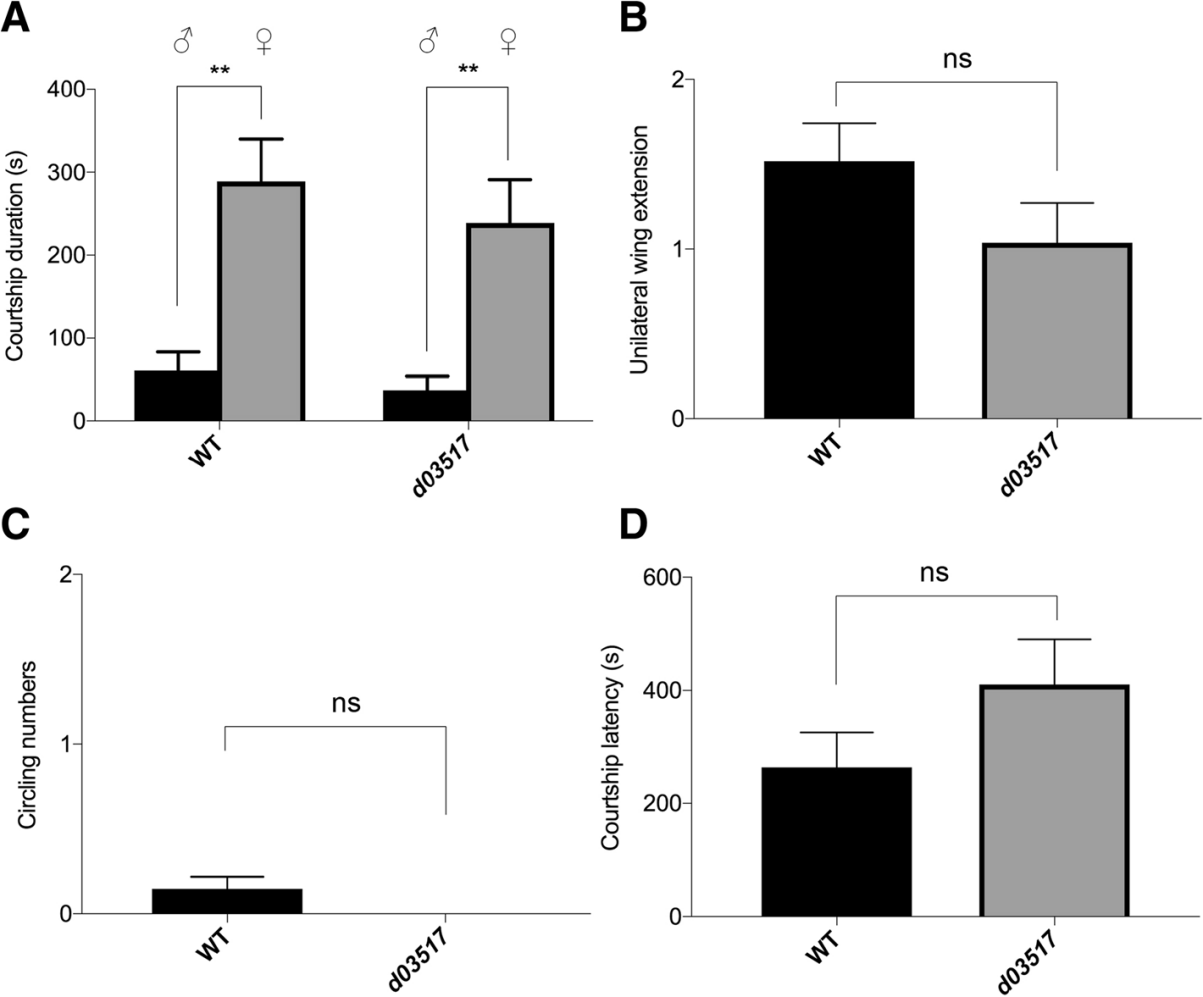
*d03517* insertion did not affect sexual behaviours. **a** Sexual discrimination assay. Both wild-type and *d03517* mutant male flies preferred to show courtship behaviours towards decapitated virgin female flies than decapitated male flies. Number of male flies tested: *wt*, *n* = 20; *d03517*, *n* = 20. Mann-Whitney U test, ^**^P < 0.01. **b-d** Male-male courtship behaviours for 15-min period. Wild-type and *d03517* mutant male flies showed very similar male-male courtship indices (Mann-Whitney U test, *P* > 0.05), including one-wing extension frequency (**b**), circling frequency (**c**), latency to courtship (**d**). “ns”, not significant, *P* > 0.05. Pairs of flies tested: *wt*, *n* = 27; *d03517*, *n* = 27. Error bars represent SEM

To distinguish among these possibilities, we assessed the ability of *d03517* mutant male flies to distinguish between males and females. A decapitated virgin female and a decapitated male were placed on different areas in a small chamber. A wild-type or a *d03517* mutant male fly was then introduced into the chamber. Like wild-type male flies (Fig. 2a), *d03517* mutant males selected the decapitated virgin female over the decapitated male for showing courtship behaviours (Fig. 2a, Additional file 5: Movie 3). This result indicates that *d03517* mutant male flies were still able to recognize sexual identities of other flies, and their sexual preference was not altered.

Above results, however, do not exclude the possibility that when only encountering a single male fly, a *d03517* mutant male fly may choose courtship over aggression leading to a decrease in aggressiveness. To address this possibility, we examined male-male courtship behaviours. A male fly was paired with another male fly, and courtship indices (i.e. unilateral wing vibration, circling frequency, and courtship latency) were analyzed. Wild-type flies showed low-frequency male-male courtship behaviours (Fig. 2b-d). Compared to wild-type male flies, *d03517* mutant male flies did not show an increase in male-male courtship behaviours (Fig. 2b-d).

We also analyzed male-female sexual behaviours. A male fly was paired with a virgin female fly, and courtship indices (i.e. unilateral wing vibration, circling frequency, courtship latency and copulation latency) were analyzed. No significant difference in male-female mating behaviours was observed between wild-type and *d03517* mutant male flies (Additional file 6: Figure S3).

In summary, *d03517* insertion decreased fly aggressiveness, but did not affect locomotor activity, olfactory avoidance response and sexual behaviours. We named the corresponding gene of this phenotype (i.e. decrease in aggressiveness) *peacefulness* (*pfs*), and *d03517* insertion is hereinafter referred to as *pfs*^d03517^.

### The *pfs* gene encodes for the fly ortholog of Mocs1

**Fig. 3:**
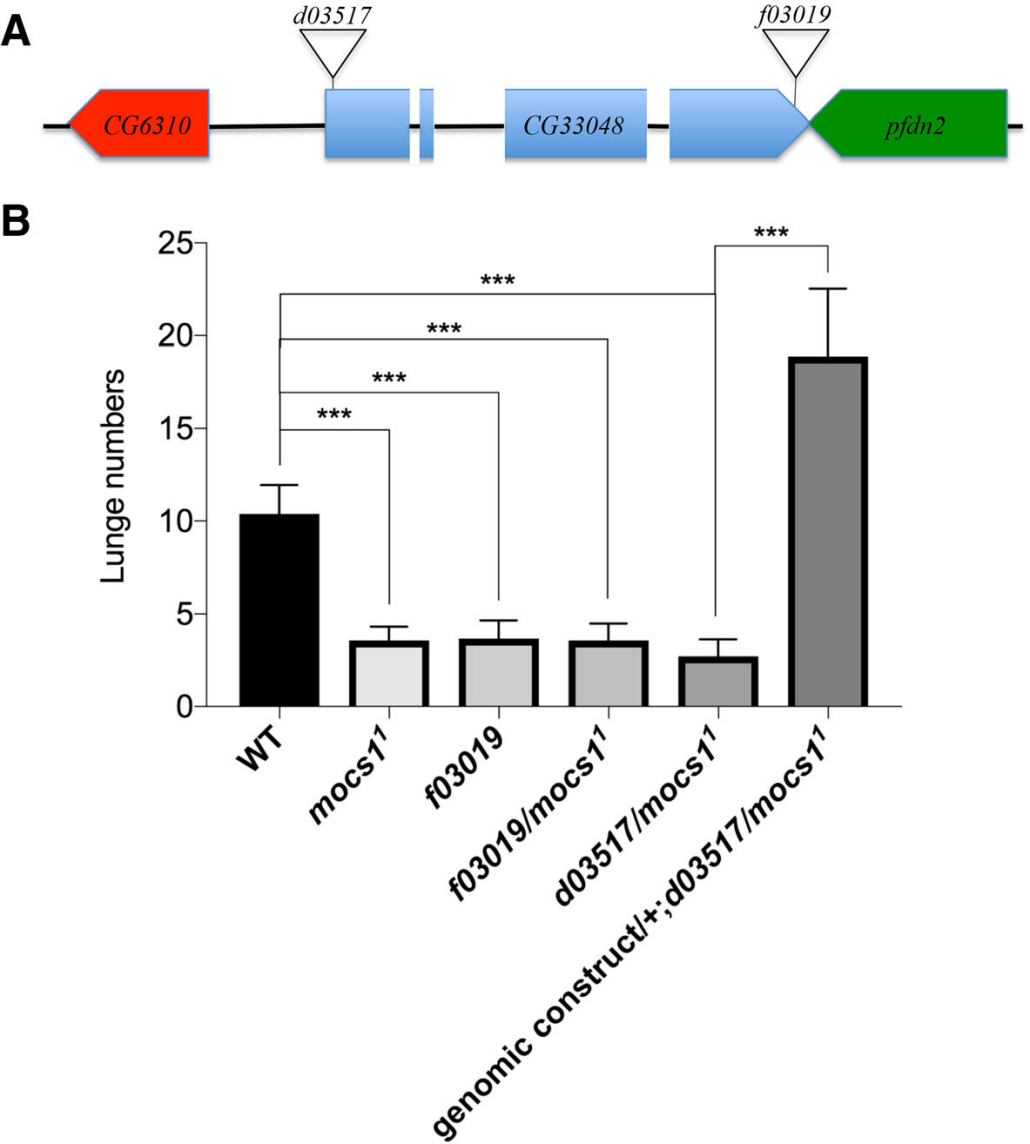
The *pfs* gene is *CG33048* that encodes for the fly ortholog of Mocs1. **a** The organization of genes near insertion sites of *d03517* and *PBac{WH}f03019 (f03019)*. *d03517* is inserted into the 1st exon of *CG33048*, 126 bp downstream of the transcription start site. *f03019* is inserted into the 4th exon of *CG33048*, 104 bp upstream of the transcription stop site. Exons and introns of *CG33048* are indicated by blue rectangles and black lines, respectively. **b** Complementation tests and transgene rescue. Behaviours of a pair of male flies for 15-min period were recorded and analyzed by the CADABRA automated analysis system [28]. All mutant alleles were backcrossed with *CS* wild-type flies for four generations and thus were in *w*^+^ background. Pairs of flies tested: *wt*, *n* = 28; *mocs1*^1^/ *mocs1*^1^, *n* = 26; *f03019/f03019*, *n* = 25; *f03019/mocs1*^1^, *n* = 21; *d03517/mocs1*^1^, *n* = 22; *genomic rescue construct/+; d03517/mocs1*^1^, *n* = 33. Kruskal-Wallis and post hoc Mann-Whitney U tests, ***P < 0.001. Error bars represent SEM

*pfs*^d03517^ is inserted into a genomic site within the first exon of the gene *CG33048* located on the 3rd chromosome (Fig. 3a) [32, 33]. *CG33048* encodes for an enzyme that is the fly ortholog of Molydenum Cofactor Protein 1 (Mocs1). In addition to *CG33048*, several other genes are also located close to the *d03517* insertion site. Since *d03517* is inserted into the first exon of *CG33048*, we performed complementation tests to examine if *d03517* is allelic to available mutations affecting *CG33048*. We firstly tested *mocs1*^1^, a partial loss-of-function mutation that decreases the enzymatic activity of Mocs1 [34, 35]. Sequencing analysis shows that *mocs1*^1^ carries a C-to-T point mutation in the coding sequence that changes Thr304 into Ile (see Materials and methods). We found that both *mocs1*^1^ homozygotes and *d03517*/*mocs1*^1^ trans-heterozygotes showed a significant decrease in the levels of intermale aggressiveness (Fig. 3b). We then examined another P-element insertion line *PBac{WH}f03019* (*f03019*) in which P-element is inserted into the 4th exon of *CG33048* [32, 33]. Similarly, a significant decrease in intermale aggressiveness was observed in *f03019* homozygotes and *f03019*/*mocs1*^1^ transheterozygotes (Fig. 3b). These results suggest strongly that the *pfs* gene is *CG33048*.

To further confirm this, we performed transgene rescue experiments. We generated transgenic flies carrying a genomic rescue construct containing entire *CG33048* sequence, and then crossed this rescue transgene into *pfs* mutant background. We found that the aggression phenotype in *pfs* mutants could be completely rescued by *CG33048* (Fig. 3b).

Taken together, these results indicate that the corresponding gene of the aggression phenotype is *CG33048* that encodes for the fly ortholog of Mocs1.

### Pfs is broadly expressed in the brain

**Fig. 4:**
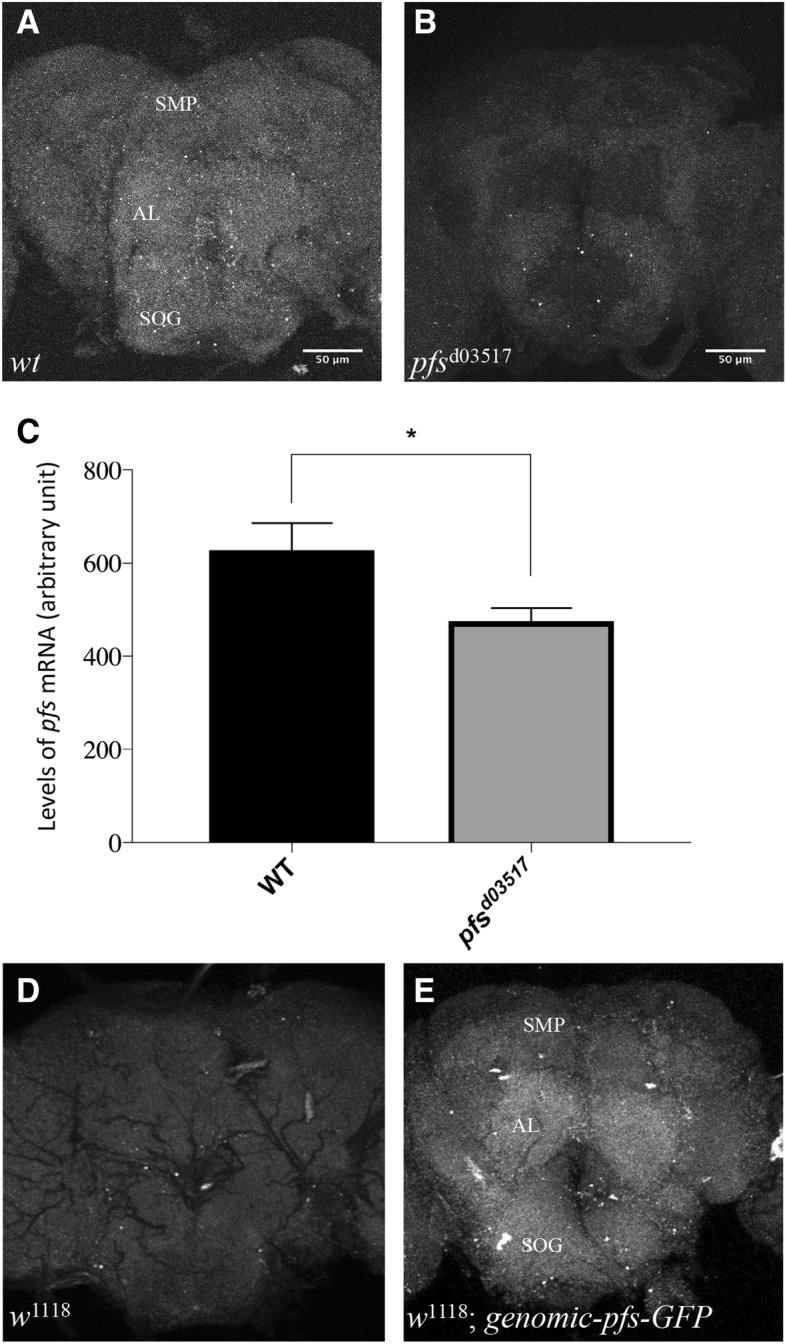
*pfs* is broadly expressed in the adult male fly brain. **a** and **b** in situ hybridization detecting *pfs* mRNAs in an adult male fly brain. **a** Wild type. **b** *pfs*^d03517^ homozygous mutant. **c** Relative expression levels of *pfs* were quantified. Compared to that in wild type, the level of *pfs* mRNAs was significantly reduced in *pfs*^d03517^ homozygous mutants. Number of flies tested: *wt*, *n* = 7; *pfs*^d03517^, *n* = 7. Student’s t-test, *P < 0.05. Error bars represent SEM. **d** and **e** Confocal sectioning of fluorescence in male adult brains of *w*^1118^ control flies (**d**) and *w*^1118^ flies carrying GFP-tagged genomic *pfs* transgene (i.e. genomic-*pfs*-GFP) (**e**). **d** Autofluorescence background in *w*^1118^ control flies. **e** Pfs-GFP was broadly expressed in the adult brain of *w*^1118^ flies carrying genomic-*pfs*-GFP. Abbreviations: SMP, superior medial protocerebrum; AL, antennal lobe; SOG, suboesophageal ganglion. Scale bar: 50 μm

We then performed in situ hybridization to examine the expression pattern of Pfs/Mocs1. We found that *pfs* mRNAs were broadly expressed throughout the brain (Fig. 4a). The intensity of staining was significantly decreased in *pfs*^d03517^ homozygous mutants, supporting the specificity of the staining (Fig. 4b and c). Within the brain, *pfs* mRNA showed higher levels of expression in superior medial protocerebrum (SMP), antennal lobe (AL), and suboesophageal ganglion (SOG) (Fig. 4a).

To examine the distribution of Pfs protein, we tagged the Pfs protein by engineering the *pfs* genomic construct that rescued the aggression phenotype (see Fig. 3b). Consistent with the results from in situ hybridization (Fig. 4a), we found that the tagged Pfs under control of the endogenous regulatory sequence showed a broad distribution in the adult brain (Fig. 4e).

### Neuronal-specific knockdown of *pfs* decreased intermale aggressiveness

**Fig. 5:**
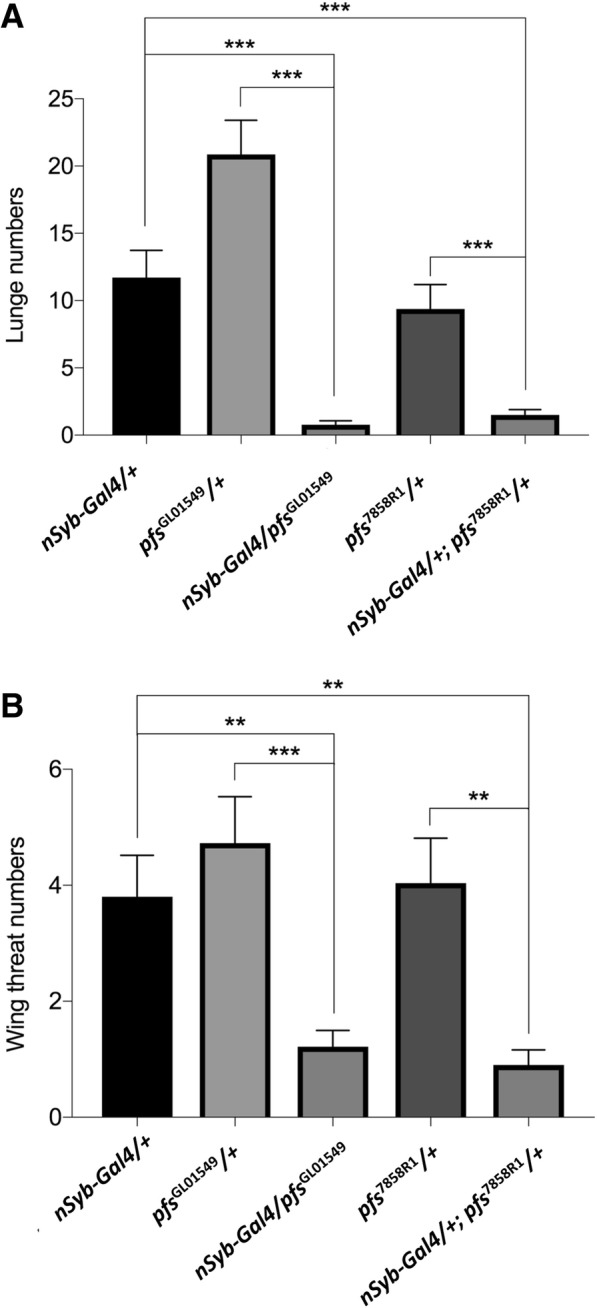
Neuronal-specific knockdown of *pfs* decreased aggressiveness. *Pfs* was knocked down in flies carrying a pan-neuronal-specific driver nSyb-Gal4 and a UAS-*pfs*-*RNAi* transgene. Two independent UAS-*pfs-RNAi* transgenes *pfs*^GL01549^ and *pfs*^7858R1^ were used in the experiments. **a** Number of lunges. **b** Number of wing threats. Behaviours of a pair of male flies for 15-min period were examined. Pairs of flies tested: *nSyb*-Gal4*/+*, *n* = 25; *pfs*^GL01549^*/+*, *n* = 22; *nSyb-*Gal4*/ pfs*^GL01549^, *n* = 23; *pfs*^7858R1^*/+*, *n* = 28; *nSyb-*Gal4*/+; pfs*^7858R1^*/+*, *n* = 20. Kruskal-Wallis and post hoc Mann-Whitney U tests, ^**^P < 0.01. ^***^P < 0.001. Error bars represent SEM

To determine if *pfs* is required in neurons for the control of aggression, we performed neuronal-specific knockdown of *pfs*. A UAS-*pfs*-*RNAi* transgene (*pfs* ^GL01549^) was expressed in all neurons under control of the neuronal-specific driver nSyb-Gal4. We found that male flies expressing this UAS-*pfs-RNAi* transgene displayed a significant decrease in aggressiveness (Fig. 5a and b). Similar results were obtained when *pfs* was knocked down by neuronal-specific expression of another independent UAS-*pfs-RNAi* transgene (*pfs*^7858R1^) (Fig. 5a and b). These results indicate an essential role for *pfs* in neurons for fly aggression.

### Overexpression of *pfs* greatly increased intermale aggressiveness

**Fig. 6:**
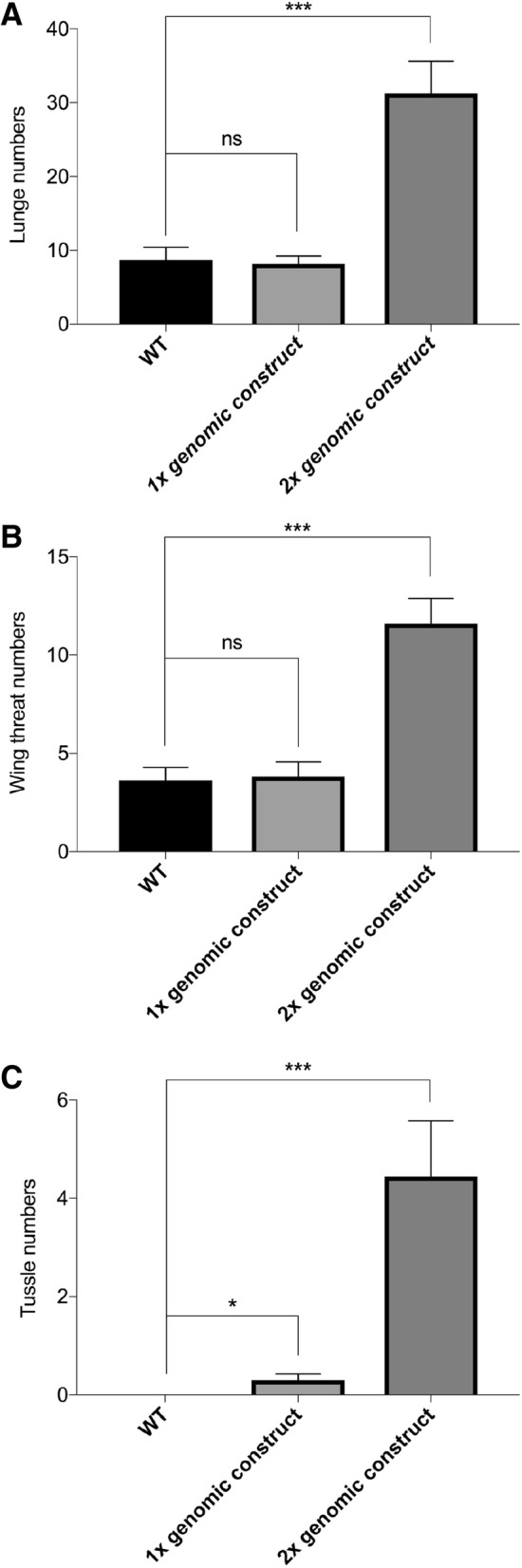
*pfs* overexpression greatly increased intermale aggressiveness. Behaviours of a pair of male flies for 15-min period were examined. **a** Number of lunges. **b** Number of wing threats. **c** Number of tussles. Introduction of one copy of the *pfs* genomic rescue construct into wild-type flies did not increase the number of lunges or wing threats, but led to a small but significant increase in tussling, an intense fighting behaviour rarely observed in wild type. When two copies of the *pfs* genomic rescue construct were introduced into wild-type flies, all agonistic behaviours were significantly increased. Pairs of flies tested: *wt*, *n* = 22; 1× genomic construct, *n* = 27; 2× genomic construct, *n* = 25. Kruskal-Wallis and post hoc Mann-Whitney U tests, ^*^P < 0.05, ^***^P < 0.001. “ns”, not significant, *P* > 0.05. Error bars represent SEM

Above results indicate a necessary role for *pfs* in the control of fly aggression. To determine if Pfs actively promotes aggressiveness, we overexpressed Pfs in flies and examined their intermale aggressive behaviours. The genomic rescue transgene containing the entire *pfs* gene was crossed into wild-type flies. Although one copy of this transgene did not significantly increase the number of lunges or wing threats (Fig. 6a and b), we found that with one copy of this transgene, there was a small but significant increase in tussling, an intense fighting behaviour that is rarely observed in wild-type flies (Fig. 6c). More strikingly, when two copies of this transgene were introduced into wild-type flies, all agonistic behaviours were greatly increased (Fig. 6a-c, compare Additional file 7: Movie S4B to S4A).

### Knocking down another component of the MoCo synthesis pathway also decreased intermale aggressiveness

**Fig. 7:**
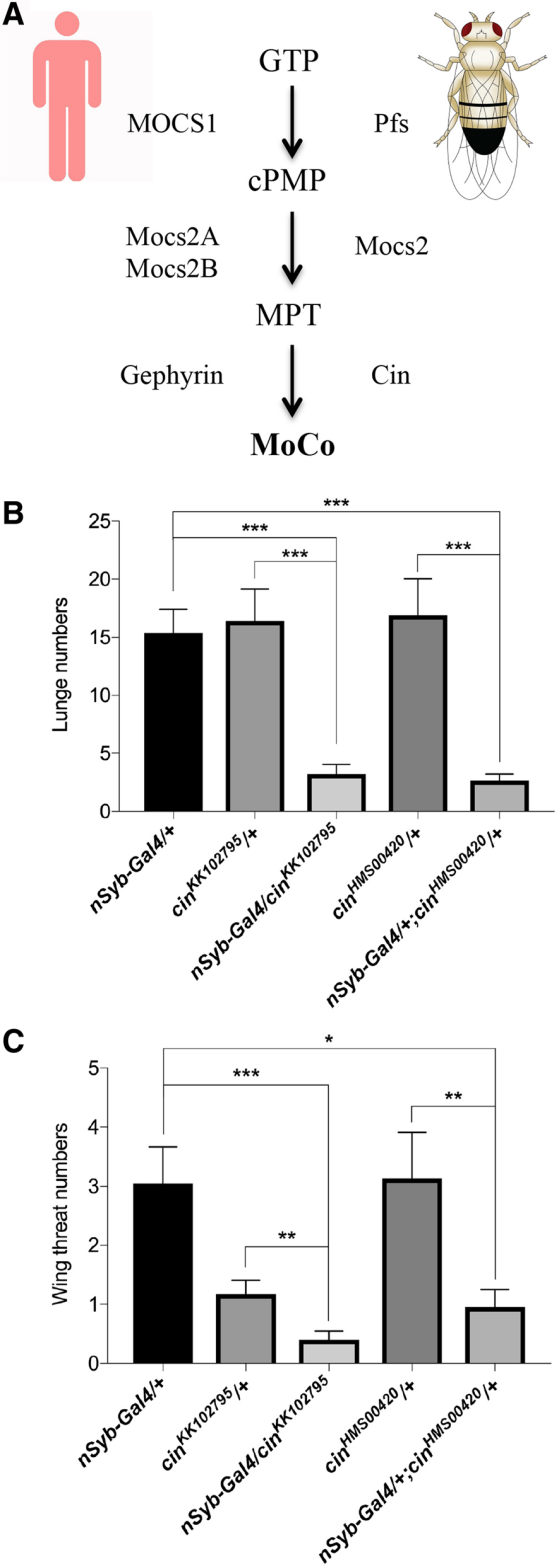
Knocking down *cin* decreased aggressiveness. **a** Schematic illustration of the MoCo biosynthesis pathway in *Drosophila* and humans. Mocs1 catalyzes the conversion of GTP to cPMP, which is converted to molybdopterin (MPT) by Mocs2 in *Drosophila* and by Mocs2A and 2B in humans. Cin or its human ortholog Gephyrin catalyzes the conversion of MPT to MoCo. **b** and **c** *cin* was knocked down in flies carrying a pan-neuronal-specific driver *nSyb*-Gal4 and a UAS-*cin*-*RNAi* transgene. Two independent UAS-*cin-RNAi* transgenes *cin*^KK102795^ and *cin*^HMS00420^ were used in the experiments. **b** Number of lunges. **c** Number of wing threats. Behaviours of a pair of male flies for 15-min period were examined. Pairs of flies tested: *nSyb-*Gal4*/+*, *n* = 21; *cin*^KK102795^*/+*, *n* = 24; *nSyb-*Gal4*/cin*^KK102795^, *n* = 25; *cin*^HMS00420^*/+*, *n* = 22; *nSyb-*Gal4*/+;cin*^HMS00420^*/+*, *n* = 23. Kruskal-Wallis and post hoc Mann-Whitney U tests, ^*^P < 0.05. ^**^P < 0.01. ^***^P < 0.001. Error bars represent SEM

Pfs may regulate intermale aggression through its function in the MoCo synthesis pathway. Alternatively, Pfs may function in a different pathway that is required for fly aggression. To distinguish among these possibilities, we tested if knocking down *cinnamon* (*cin*), encoding for another enzyme catalyzing the last step in the MoCo synthesis pathway (Fig. 7a) [22], causes a *pfs*-like aggression phenotype.

The expression of *cin* was knocked down in flies by expressing a UAS-*cin-RNAi* transgene (i.e. *cin*^KK102795^) under control of the neuronal-specific driver nSyb-Gal4. Compared to control flies carrying either the driver or the UAS-*cin-RNAi* alone, *cin* knockdown flies showed a significant decrease in aggressiveness (Fig. 7b and c). We also performed knockdown by using a different UAS-*cin-RNAi* transgene (i.e. *cin*^HMS00420^). A similar decrease in aggressiveness was observed (Fig. 7b and c).

To determine potential effects of reducing *cin* on physical capabilities of knockdown flies, we examined fly climbing ability and locomotor activity. No significant difference in climbing ability was observed when knockdown flies carrying both nSyb-Gal4 driver and UAS-*cin-RNAi* (i.e. *cin*^KK102795^ or *cin*^HMS00420^) were compared to control flies carrying nSyb-Gal4 driver or UAS-*cin-RNAi* only (Additional file 8: Figure S4A).

We also examined fly locomotion over 15-min period. For knockdown using UAS-*cin*^HMS00420^, no significant difference in total distance of movements was observed when comparing knockdown flies carrying both nSyb-Gal4 driver and UAS-*cin*^HMS00420^ to control flies carrying nSyb-Gal4 driver or UAS-*cin*^HMS00420^ only (Additional file 8: Figure S4B). For knockdown using UAS-*cin*^KK102795^, the travel distance of knockdown flies carrying both nSyb-Gal4 driver and UAS-*cin*^KK102795^ is lower than that of control flies carrying nSyb-Gal4 driver only, but is not significantly different from that of control flies carrying UAS-*cin*^KK102795^ only (Additional file 8: Figure S4B). Those results argue against that the decrease in the levels of aggressiveness in *cin* knockdown flies was caused by impaired physical capabilities.

Taken together, these results support that Pfs/Mocs1 regulates aggression through its action in the MoCo biosynthesis pathway.

## Discussion

In this study, we identify Pfs as a novel and important player in the control of intermale aggression in *Drosophila*. Mutations in *pfs* decreased intermale fly aggressiveness, but did not affect locomotor activity, climbing ability, olfactory avoidance response and sexual behaviours. Like *pfs* mutations, knocking down another component (i.e. Cin) of the MoCo synthesis pathway also decreased intermale aggressiveness, supporting a necessary role for Pfs/Mocs1 in the MoCo biosynthesis pathway for fly aggressiveness. That overexpression of Pfs caused a dramatic increase in intermale aggressiveness suggests strongly that Pfs/Mocs1 and the MoCo synthesis pathway actively promote intermale aggression in *Drosophila*.

We propose that Pfs/Mocs1 controls fly aggression by regulating the synthesis of MoCo, which in turn modulates the activity of MoCo-dependent molybdoenzymes. The MoCo biosynthesis pathway is conserved throughout evolution [22]. MoCo synthesis involves multiple steps that convert guanosine triphosphate (GTP) to MoCo. Mocs1 catalyzes the first step that is the conversion of GTP to cyclic pyranopterin monophosphate (cPMP). cPMP is then converted to molybdopterin (MPT) dithiolate by MPT synthase, which consists of two subunits Mocs2A and Mocs2B. The final step is catalyzed by Gephyrin, leading to the conversion of MPT to MoCo. MoCo forms the active site of all eukaryotic molybdenum-dependent molybdoenzymes such as sulphite oxidase, xanthine oxidase/dehydrogenase and aldehyde oxidase [23]. Interestingly, it is reported that allopurinol, an inhibitor of xanthine oxidase, displays anti-aggressive effects, and could effectively treat dementia and schizophrenia patients associated with escalated aggression [24–27]. Thus, Pfs/Mocs1-dependent MoCo pathways may control aggression across phylogeny.

Pfs/Mocs1 may control aggression by regulating metabolic activities in the brain. MoCo-dependent molybdoenzymes are involved in the regulation of a number of metabolic activities [23]. Sulphite oxidase is required for the degradation of sulphur-containing amino acids and lipids [36]. Xanthine oxidase mediates the catabolism of purines by converting hypoxanthine to uric acid [37]. And aldehyde oxidase is involved in the catabolism of bioamines such as serotonin and dopamine [38]. Together, these molybdoenzymes may modulate the metabolic state within the brain for promoting aggression. A link between glucose metabolism and aggressiveness has been reported recently [39]. By manipulating oxidation phosphorylation in honeybee and *Drosophila*, Robinson and coworkers show that aerobic glycolysis increases aggressiveness. Similarly, we speculate that Pfs/Mocs1 regulates MoCo-dependent molybdoenzymes through MoCo synthesis, which in turn modulate metabolic plasticity in the brain for the control of aggression.

Pfs/Mocs1-dependent MoCo pathways may also promote aggressiveness by increasing oxidative stress. Both xanthine oxidase and aldehyde oxidase catalyze the reactions leading to the generation of reactive oxygen species (ROS), such as hydrogen peroxide and superoxide ion [40, 41]. Oxidative stress caused by the accumulation of ROS, has been linked to anxiety and aggression in animal models [42, 43]. For instance, mouse defective in superoxide dismutase 1 (Sod1), an enzyme with antioxidant activity, displays a dramatic increase in aggressiveness [43].

MoCo deficiency (MOCOD) is a rare and severe disease in humans [44]. Patients with MOCOD display severe neurological symptoms, such as intellectual disability, autism, seizures, feeding difficulties, and neurodevelopmental abnormalities. It is suggested that neural damages are mainly due to sulfite oxidase deficiency and the accumulation of toxic levels of sulphite [45]. Over 50% of MoCo deficiency in humans are due to mutations in the MOCS1A open reading frame [46], which mostly result in early death of children [47]. By contrast, we did not observe any developmental defects in fly *pfs* mutants. One likely explanation is that *pfs* alleles are not null, and thus do not completely eliminate the activity of MoCo-dependent molybdoenzymes. Alternatively or additionally, flies may be more resistant to the accumulation of toxic metabolic intermediates due to the decrease in the activities of MoCo-dependent molybdoenzymes.

Our results showing the aggression phenotype caused by manipulating the MoCo biosynthesis pathway in *Drosophila*, together with observed anti-aggressive effects by the inhibition of MoCo-dependent enzymes in human patients, support the existence of a novel and evolutionarily conserved MoCo-dependent mechanism for the control of aggression. A number of neurological and psychiatric disorders, such as schizophrenia, dementia and Alzheimer’s disease, show a substantial association with abnormal aggressiveness [14, 48]. It would be interesting to determine if patients with these disorders show elevated levels of MoCo and/or MoCo-dependent molybdoenzymes. Targeting Pfs/Mocs1 and molybdoenzymes may thus allow the development of novel therapeutic strategies to treat diseases associated with escalated aggression.

## Materials and methods

### Genetics and rearing conditions

P-element insertion lines *P{XP}d03517* and *PBac{WH}f03019* were obtained from the Exelixis collection at Harvard. *mocs1*^1^, UAS*-pfs-RNAi*-*GL01549*, and UAS*-cin-RNAi-HMS00420* lines were obtained from Bloomington Stock Center. The UAS*-pfs-RNAi-7858R1* line was obtained from National Institute of Genetics Fly Stock Center in Japan. The UAS-*cin-RNAi-KK102795* line was obtained from the Vienna Drosophila Resource Center. To eliminate the effects of different genetic backgrounds on fly behaviours, all *pfs* mutant alleles were backcrossed with *Canton-S* (*CS*) wild-type flies for 4 generations and were in *w*^+^ background. *CS* flies were used as wild-type controls in the experiments. For knockdown experiments, female flies carrying the Gal4 driver were crossed with male flies carrying the UAS-*RNAi* transgene. The progeny male flies carrying both Gal4 and UAS-*RNAi* transgenes were then compared to male flies carrying Gal4 driver or UAS-*RNAi* transgene only. Flies were reared on standard corn meal at 25 °C and 50–60% humidity with 12 h light-dark cycle.

For rescue experiments, the genomic fragment containing the entire coding sequence (2823 bp) of the *CG33048* gene, the 1019 bp sequence upstream of *CG33048* and the 755 bp sequence downstream of *CG33048*, was amplified by PCR with two primers 5’ CTCCGAGCGGAGACTCTAGCGCTAGCCTCTGTGTACTGCACCGTGTA 3′ and 5’ CTCACCATGGATCCAGATCCACTAGTGGGCCCAAAGATGGATGACA 3′. The resulting PCR fragment and the pJFRC-MUH-eGFP vector linearized with Nhe1 and Spe1, were ligated together using In-Fusion HD Cloning Plus Kits (Takara Bio UAS Inc.) to generate genomic rescue construct for making transgenic flies. Genetic crosses were then performed to introduce the *CG33048* genomic rescue construct into *mocs1* trans-heterozygous mutants (i.e. *pfs*^d03517^*/mocs1*^1^). To overexpress Pfs, wild-type flies carrying one or two copies of the *CG33048* genomic rescue construct were generated.

To tag the *pfs* coding sequence in above genomic rescue construct with GFP, the genomic rescue construct was used as templates for PCR amplification. Two primers 5’ CTCCGAGCGGAGACTCTAGCGCTAGCCTCTGTGTACTGCACCGTGTA 3′ and 5’ CTCACCATGGATCCAGATCCACTAGTTTCGACTTCTGTAACTATCC 3′ were used for amplifying the entire upstream regulatory sequence and the *pfs* coding sequence before the stop codon. The resulting PCR fragment and the pJFRC-MUH-eGFP vector linearized with Nhe1 and Spe1, were ligated together using In-Fusion HD Cloning Plus Kits (Takara Bio UAS Inc.) to generate the genomic-*pfs*-GFP construct. The genomic *pfs*-GFP construct differs from the rescue construct only in the sequence downstream from the coding sequence; while the genomic rescue construct contains 755 bp genomic sequence downstream of the *pfs* coding sequence, the genomic *pfs*-GFP construct contains an in-frame fusion between the last codon of the *pfs* coding sequence and the GFP sequence.

To identify mutation sites in the *mocs1*^1^ allele, genomic DNA was isolated from homozygous *mocs1*^1^ mutant flies and used as templates for PCR amplification. The resulting fragment containing the entire sequence (2823 bp) of *pfs* was sequenced completely, which was then compared to that of wild-type *pfs* genomic sequence. A C-to-T missense mutation in the coding sequence that changes Thr304 to Ile in Pfs/Mocs1 protein was identified in the genomic DNA of the *mocs1*^1^ allele.

### Aggression assays

Newly emerged male flies were collected and isolated in 2 ml Eppendorf tubes containing 1 ml fly food for 5 to 7 days before behavioural experiments. Male flies of same age with similar body size were selected for behavioural assays. Aggressive behaviours were examined similarly as described previously [28]. Briefly, two male flies were gently aspirated into a fighting chamber at 25 °C with 50–60% humidity. Their behaviours were immediately recorded with a CCD camera for 15-min period. The movies were then analyzed by using the CADABRA (Caltech Automated Drosophila Aggression-Courtship Behavioral Repertoire Analysis) automated analysis system [28]. For examining aggressive behaviours between a wild-type and a mutant male fly, wild-type flies were labeled with color paint.

To determine the potential dominance, wild-type and mutant male flies were anesthetised by CO_2_, and marked on thorax with yellow and white acrylic paints, respectively. Flies were allowed for recovery at least 24 h before behavioural assays. A wild-type male fly was paired with a mutant male fly and introduced into a small chamber containing a food patch in the center similarly as described previously [49]. A score of 1 was given if a fly (e.g. wild type) successfully occupied the central food patch after 10-min period. A score of 0 indicates that the fly did not occupy the central food patch after 10-min period. Successful occupancy of the central food patch is considered as an indication of dominance.

### Analysis of *pfs* expression in male adult brains

For fluorescence in situ hybridization, custom Stellaris® FISH probes were designed to detect *pfs*/*mocs1* mRNAs by utilizing the Stellaris® FISH Probe Designer (Biosearch Technologies, Inc., Petaluma, CA) available online at www.biosearchtech.com/stellarisdesigner. The probes were conjugated to the Quasar670 dye and used in FISH assays as described previously [50]. Confocal microscopy was performed by using Olympus laser scanning microscope FV1000. For comparing the relative levels of *pfs* mRNAs in wild-type and *pfs* mutant flies, fluorescent intensities in the central brain region were measured.

To examine the expression pattern of genomic-*pfs* tagged with GFP, adult heads were dissected in phosphate buffer. The brains were immediately mounted on glass slides in phosphate buffer and imaged with confocal microscopy using Olympus laser scanning microscope FV1000.

### Male-male courtship assay

To examine male-male courtship behaviours, a pair of male flies with same genotype were introduced into a rectangular chamber. Their behaviours were recorded for 15 min. Unilateral wing extensions and circling numbers were quantified by using the CADABRA automated analysis system. Courtship latency was quantified manually.

### Sexual discrimination assay

For each experiment, a CS wild-type virgin female fly and a CS wild-type male fly were decapitated. The decapitated flies were placed to different areas in a rectangular chamber. A test male fly (wild-type or mutant) was then introduced into the chamber. The time that the test male fly showed courtship behaviours towards the decapitated female or the decapitated male fly was quantified.

### Statistical analysis

Statistical analysis was performed using GraphPad Prism 7 software. Before data analysis, their normality distributions were examined. Nonparametric tests were performed for data not normally distributed. For comparing more than two genotypes, a Kruskal-Wallis test was performed. If the null hypothesis (i.e. means of all genotypes were the same) was rejected (*P* < 0.05), we performed multiple Mann-Whitney U tests between a pair of interest to assess whether the means of the two genotypes were significantly different. For comparing two independent groups, an unpaired Mann-Whitney U test was performed. Student’s t-test was performed for data normally distributed.

## Additional files

Additional file 1: **Movie S1A.** and **S1B.** Video clips showing aggressive behaviours of wild-type (S1A) and *d03517* mutant (S1B) male flies, related to Fig. 1a and b. Male flies of the same genotype were paired together. (ZIP 10240 kb)

Additional file 2: **Movie S2.** A video clip showing aggressive behaviours of a wild-type male fly paired with a *d03517* mutant male fly, related to Fig. 1d. The thorax of wild-type male fly was painted with a yellow acrylic paint dot, and the thorax of *d03517* male fly was painted with a white acrylic paint dot. Wild-type flies showed much higher aggressiveness, and also occupied the food patch much more successfully. (MP4 2580 kb)

Additional file 3: **Figure S1.** *d03517* insertion did not affect physical capabilities. (A) Locomotor activity for 15-min period. A wild-type male fly was paired with a *d03517* mutant male fly. Total travel distance for 15-min period was quantified. No significant difference between wild-type and *d03517* male flies was observed (Mann-Whitney U test, “ns”, not significant, *P* > 0.05). Number of flies tested: wild type, *n* = 46; *d03517/ d03517*, 46. Methods: A pair of male flies were introduced into a chamber. Their behaviours were videotaped for 15 minutes. Their movements within 15-minute period were analyzed and quantified using the CADABRA automated analysis system. (B) Climbing test. The percentage of flies that crossed the 10-cm mark after 15-s climbing were quantified. Number of trials: *wt*, *n* = 30; *d03517/ d03517*, *n* = 30. No significant difference between wild-type and *d03517* mutant flies was observed (Mann-Whitney U test, “ns”, not significant, *P* > 0.05). Error bars represent SEM. Methods: For each trial, 10-12 male flies (5-7 days after eclosion) were transferred into atransparent tube. The flies were allowed to recover for 1 hour. The tube was then tapped three times to force flies down at the bottom of the tube. Their climbing behaviors were videotaped. The percentage of flies that crossed the 10-cm mark after 15 seconds was quantified. The experiment was repeated 3 times for each tube. For each genotype, about 86 to 122 flies were tested. (PDF 105 kb)

Additional file 4: **Figure S2. ***d03517* insertion did not affect olfactory avoidance response. (A) T-maze apparatus used for testing olfactory avoidance response. The apparatus consists of two separate compartments. One compartment is used for fly habituation following their introduction into the apparatus. The second compartment connects to two plastic tubes. One tube is empty, and another tube is filled with benzaldehyde. (B) Olfactory avoidance responses by wild-type and *d03517* mutant flies. No significant difference between wild-type and *d03517* male flies was observed (Mann-Whitney U test, “ns”, not significant, *P* > 0.05). Number of tests per genotype: wt, *n* = 12; *d03517/d03517*, *n* = 7. For each test, 10–20 flies were examined. Error bars represent SEM. Methods: Prior to the experiments, flies were deprived of food for 3-6 hours. They were then introduced into a T-maze apparatus containing two compartments (Supplementary Fig. S2A). The first compartment is for fly habituation. The second compartment connects to two plastic tubes. One tube is empty. Another tube has a cotton ball containing 1ml of benzaldehyde, a strong fruit fly repellent, at the open end. For each experiment, 10-20 flies were gently introduced into the apparatus. Flies were kept in the first compartment for 90 seconds, and then allowed to move into the second compartment for 120 seconds. Number of flies that moved into benzaldehyde-containing tube or empty tube were counted. Smell index 10.1186/s13041-018-0417-0 was then calculated as follows: Smell index= (Number of flies in empty tube-number of flies in benzaldehyde tube)/(Total number of flies). (PDF 220 kb)

Additional file 5: **Movie S3.** A video clip showing a d03517 mutant male fly selected a decapitated female fly over a decapitated male fly for courtship, related to Fig. 2a. (MP4 1487 kb)

Additional file 6: **Figure S3.** Male-female courtship behaviours for 15-min period. Wild-type and *d03517* mutant male flies showed very similar male-female courtship indices (Mann-Whitney U test, *P* > 0.05), including one-wing extensions (A), circling frequency (B), latency to courtship (C), and latency to copulation (D). Number of flies tested: wt, *n* = 20; *d03517/ d03517*, *n* = 20. Error bars represent SEM. Methods: To examine male-female courtship behaviours, a CS wild-type virgin female fly was paired with a wild-type or a mutant male fly, and introduced into a rectangular chamber. Their behaviours were recorded for 15 minutes. Unilateral wing extensions and circling numbers were quantified by using the CADABRA automated analysis system. Courtship latency and copulation latency were quantified manually. (PDF 166 kb)

Additional file 7: **Movie S4A.** and **S4B.** Video clips showing aggressive behaviours of wild-type male flies (4A) and male flies overexpressing *pfs* (4B), related to Fig. 6. Male flies of the same genotype were paired together. *pfs* overexpression greatly increased fly aggressiveness. (ZIP 8304 kb)

Additional file 8: **Figure S4.** *cin* knockdown did not affect physical capabilities. (A) Climbing test. The percentage of flies that crossed the 10-cm mark after 15-s climbing was quantified. Number of trials: *nSyb*-Gal4/+, *n* = 27; *cinKK102795/+*, *n* = 33; *nSyb*-Gal4/*cin*KK102795, *n* = 36; *cin*HMS00420/+, *n* = 24; *nSyb*-Gal4/+;*cin*HMS00420/+, *n* = 24. No significant difference was observed between knockdown flies and control flies (Kruskal-Wallis and post hoc Mann-Whitney U tests, “ns”, not significant, *P* > 0.05). (B) Locomotor activity for 15-min period. Total travel distance for 15-min period was quantified. Number of individual flies tested: *nSyb*-Gal4/+, *n* = 42; *cin*KK102795/+, *n* = 48; *nSyb*-Gal4/*cin*KK102795, *n* = 50; *cin*HMS00420/+, *n* = 44; *nSyb*-Gal4/+;*cin*HMS00420/+, *n* = 46. Kruskal-Wallis and post hoc Mann-Whitney U tests, ***P < 0.001. “ns”, not significant, *P* > 0.05. Error bars represent SEM. (PDF 180 kb)

## Abbreviations

AL: Antennal lobe
CADABRA: Caltech Automated Drosophila Aggression-Courtship Behavioral Repertoire Analysis
Cin: Cinnamon
MoCo: Molybdenum cofactor
Mocs1: Molybdenum cofactor synthesis 1 protein
Pfs: Peacefulness
SMP: Superior medial protocerebrum
SOG: Suboesophageal ganglion

## References

[1] Kravitz EA, Huber R (2003). Aggression in invertebrates. Curr Opin Neurobiol.

[2] Tecott LH, Barondes SH (1996). Genes and aggressiveness. Behavioral genetics. Curr Biol.

[3] Loeber R, Hay D (1997). Key issues in the development of aggression and violence from childhood to early adulthood. Annu Rev Psychol.

[4] Hoffmann AA (1990). The influence of age and experience with conspecifics on territorial behavior inDrosophila melanogaster. J Insect Behav.

[5] Matsumoto K, Pinna G, Puia G, Guidotti A, Costa E (2005). Social isolation stress-induced aggression in mice: a model to study the pharmacology of neurosteroidogenesis. Stress.

[6] Wang L, Dankert H, Perona P, Anderson DJ (2008). A common genetic target for environmental and heritable influences on aggressiveness in Drosophila. Proc Natl Acad Sci U S A.

[7] Kravitz EA, Fernandez Mde L (2015). Aggression in Drosophila. Behav Neurosci.

[8] Barr CS, Driscoll C (2014). Neurogenetics of aggressive behavior: studies in primates. Curr Top Behav Neurosci.

[9] Takahashi A, Miczek KA (2014). Neurogenetics of aggressive behavior: studies in rodents. Curr Top Behav Neurosci.

[10] Sturtevant AH (1915). Experiments on sex recognition and the problem of sexual selection in Drosoophilia. J Anim Behav.

[11] Jacobs ME (1960). Influence of light on mating of Drosophila melanogaster. Ecology.

[12] Hoffmann AA (1987). A laboratory study of male territoriality in the sibling species Drosophila melanogaster and D. simulans. Anim Behav.

[13] Chen S, Lee AY, Bowens NM, Huber R, Kravitz EA (2002). Fighting fruit flies: a model system for the study of aggression. Proc Natl Acad Sci U S A.

[14] Swann AC (2003). Neuroreceptor mechanisms of aggression and its treatment. J Clin Psychiatry.

[15] Hoyer SC, Eckart A, Herrel A, Zars T, Fischer SA, Hardie SL, Heisenberg M (2008). Octopamine in male aggression of Drosophila. Curr Biol.

[16] Zhou C, Rao Y, Rao Y (2008). A subset of octopaminergic neurons are important for Drosophila aggression. Nat Neurosci.

[17] Dierick HA, Greenspan RJ (2007). Serotonin and neuropeptide F have opposite modulatory effects on fly aggression. Nat Genet.

[18] Alekseyenko OV, Chan YB, Li R, Kravitz EA (2013). Single dopaminergic neurons that modulate aggression in Drosophila. Proc Natl Acad Sci U S A.

[19] Edwards AC, Ayroles JF, Stone EA, Carbone MA, Lyman RF, Mackay TF (2009). A transcriptional network associated with natural variation in Drosophila aggressive behavior. Genome Biol.

[20] Katsouni E, Sakkas P, Zarros A, Skandali N, Liapi C (2009). The involvement of substance P in the induction of aggressive behavior. Peptides.

[21] Asahina K, Watanabe K, Duistermars BJ, Hoopfer E, Gonzalez CR, Eyjolfsdottir EA, Perona P, Anderson DJ (2014). Tachykinin-expressing neurons control male-specific aggressive arousal in Drosophila. Cell.

[22] Mendel RR, Leimkuhler S (2015). The biosynthesis of the molybdenum cofactors. J Biol Inorg Chem.

[23] Schwarz G, Mendel RR, Ribbe MW (2009). Molybdenum cofactors, enzymes and pathways. Nature.

[24] Lara DR, Belmonte-de-Abreu P, Souza DO (2000). Allopurinol for refractory aggression and self-inflicted behaviour. J Psychopharmacol.

[25] Lara DR, Brunstein MG, Ghisolfi ES, Lobato MI, Belmonte-de-Abreu P, Souza DO (2001). Allopurinol augmentation for poorly responsive schizophrenia. Int Clin Psychopharmacol.

[26] Lara DR, Cruz MR, Xavier F, Souza DO, Moriguchi EH (2003). Allopurinol for the treatment of aggressive behaviour in patients with dementia. Int Clin Psychopharmacol.

[27] Carr CN, Straley CM, Baugh TB (2017). Allopurinol for the treatment of refractory aggression: a case series. Pharmacotherapy.

[28] Dankert H, Wang L, Hoopfer ED, Anderson DJ, Perona P (2009). Automated monitoring and analysis of social behavior in Drosophila. Nat Methods.

[29] Fernandez MP, Chan YB, Yew JY, Billeter JC, Dreisewerd K, Levine JD, Kravitz EA (2010). Pheromonal and behavioral cues trigger male-to-female aggression in Drosophila. PLoS Biol.

[30] Wang L, Anderson DJ (2010). Identification of an aggression-promoting pheromone and its receptor neurons in Drosophila. Nature.

[31] Liu W, Liang X, Gong J, Yang Z, Zhang YH, Zhang JX, Rao Y (2011). Social regulation of aggression by pheromonal activation of Or65a olfactory neurons in Drosophila. Nat Neurosci.

[32] Thibault ST, Singer MA, Miyazaki WY, Milash B, Dompe NA, Singh CM, Buchholz R, Demsky M, Fawcett R, Francis-Lang HL (2004). A complementary transposon tool kit for Drosophila melanogaster using P and piggyBac. Nat Genet.

[33] Bellen HJ, Levis RW, He Y, Carlson JW, Evans-Holm M, Bae E, Kim J, Metaxakis A, Savakis C, Schulze KL (2011). The Drosophila gene disruption project: progress using transposons with distinctive site specificities. Genetics.

[34] Keller EC, Glassman E (1964). A Third Locus (Lxd) affecting xanthine dehydrogenase in Drosophila melanogaster. Genetics.

[35] Schott DR, Baldwin MC, Finnerty V (1986). Molybdenum hydroxylases in Drosophila. III. Further characterization of the low xanthine dehydrogenase gene. Biochem Genet.

[36] Kappler U, Enemark JH (2015). Sulfite-oxidizing enzymes. J Biol Inorg Chem.

[37] Agarwal A, Banerjee A, Banerjee UC (2011). Xanthine oxidoreductase: a journey from purine metabolism to cardiovascular excitation-contraction coupling. Crit Rev Biotechnol.

[38] Beedham C, Peet CF, Panoutsopoulos GI, Carter H, Smith JA (1995). Role of aldehyde oxidase in biogenic amine metabolism. Prog Brain Res.

[39] Li-Byarlay H, Rittschof CC, Massey JH, Pittendrigh BR, Robinson GE (2014). Socially responsive effects of brain oxidative metabolism on aggression. Proc Natl Acad Sci U S A.

[40] Battelli MG, Polito L, Bortolotti M, Bolognesi A (2016). Xanthine oxidoreductase-derived reactive species: physiological and pathological effects. Oxidative Med Cell Longev.

[41] Kucukgoze G, Terao M, Garattini E, Leimkuhler S (2017). Direct comparison of the enzymatic characteristics and superoxide production of the four aldehyde oxidase enzymes present in mouse. Drug Metab Dispos.

[42] Bouayed J, Rammal H, Soulimani R (2009). Oxidative stress and anxiety: relationship and cellular pathways. Oxidative Med Cell Longev.

[43] Garratt M, Brooks RC (2015). A genetic reduction in antioxidant function causes elevated aggression in mice. J Exp Biol.

[44] Schwarz G (2016). Molybdenum cofactor and human disease. Curr Opin Chem Biol.

[45] Leimkuhler S, Charcosset M, Latour P, Dorche C, Kleppe S, Scaglia F, Szymczak I, Schupp P, Hahnewald R, Reiss J (2005). Ten novel mutations in the molybdenum cofactor genes MOCS1 and MOCS2 and in vitro characterization of a MOCS2 mutation that abolishes the binding ability of molybdopterin synthase. Hum Genet.

[46] Reiss J, Johnson JL (2003). Mutations in the molybdenum cofactor biosynthetic genes MOCS1, MOCS2, and GEPH. Hum Mutat.

[47] Carmi-Nawi N, Malinger G, Mandel H, Ichida K, Lerman-Sagie T, Lev D (2011). Prenatal brain disruption in molybdenum cofactor deficiency. J Child Neurol.

[48] Haller J, Kruk MR (2006). Normal and abnormal aggression: human disorders and novel laboratory models. Neurosci Biobehav Rev.

[49] Ramin M, Domocos C, Slawaska-Eng D, Rao Y (2014). Aggression and social experience: genetic analysis of visual circuit activity in the control of aggressiveness in Drosophila. Mol Brain.

[50] Raj A, van den Bogaard P, Rifkin SA, van Oudenaarden A, Tyagi S (2008). Imaging individual mRNA molecules using multiple singly labeled probes. Nat Methods.

