# Supplementary figures and images for: The peacefulness gene promotes aggression in Drosophila
Source: Mol Brain. 2019 Jan 3;12:1. doi: 10.1186/s13041-018-0417-0 (PMC6318936; doi:10.1186/s13041-018-0417-0)

A

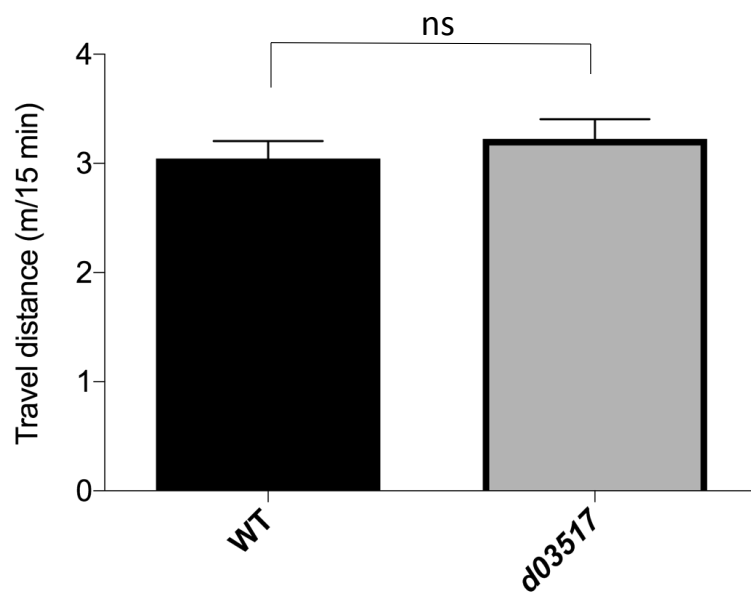

B

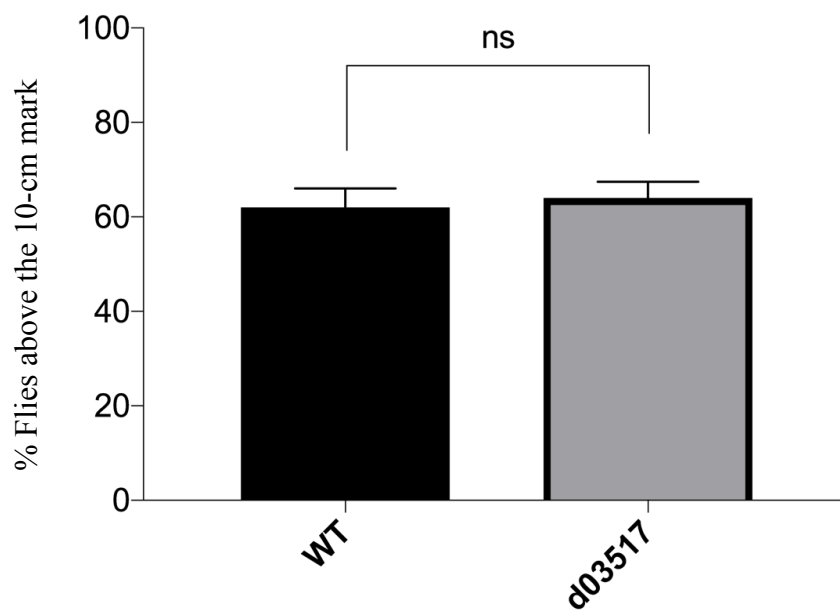

Supplementary Figure S1

Supplement: Supplementary file 3 — Figure S1. d03517 insertion did not affect physical capabilities. (A) Locomotor activity for 15-min period. A wild-type male fly was paired with a d03517 mutant male fly. Total travel distance for 15-min period was quantified. No significant difference between wild-type and d03517 male flies was observed (Mann-Whitney U test, “ns”, not significant, P > 0.05). Number of flies tested: wild type, n = 46; d03517/ d03517, 46. Methods: A pair of male flies were introduced into a chamber. Their behaviours were videotaped for 15 minutes. Their movements within 15-minute period were analyzed and quantified using the CADABRA automated analysis system. (B) Climbing test. The percentage of flies that crossed the 10-cm mark after 15-s climbing were quantified. Number of trials: wt, n = 30; d03517/ d03517, n = 30. No significant difference between wild-type and d03517 mutant flies was observed (Mann-Whitney U test, “ns”, not significant, P > 0.05). Error bars represent SEM. Methods: For each trial, 10-12 male flies (5-7 days after eclosion) were transferred into atransparent tube. The flies were allowed to recover for 1 hour. The tube was then tapped three times to force flies down at the bottom of the tube. Their climbing behaviors were videotaped. The percentage of flies that crossed the 10-cm mark after 15 seconds was quantified. The experiment was repeated 3 times for each tube. For each genotype, about 86 to 122 flies were tested. (PDF 105 kb) [file 13041_2018_417_MOESM3_ESM.pdf]

A

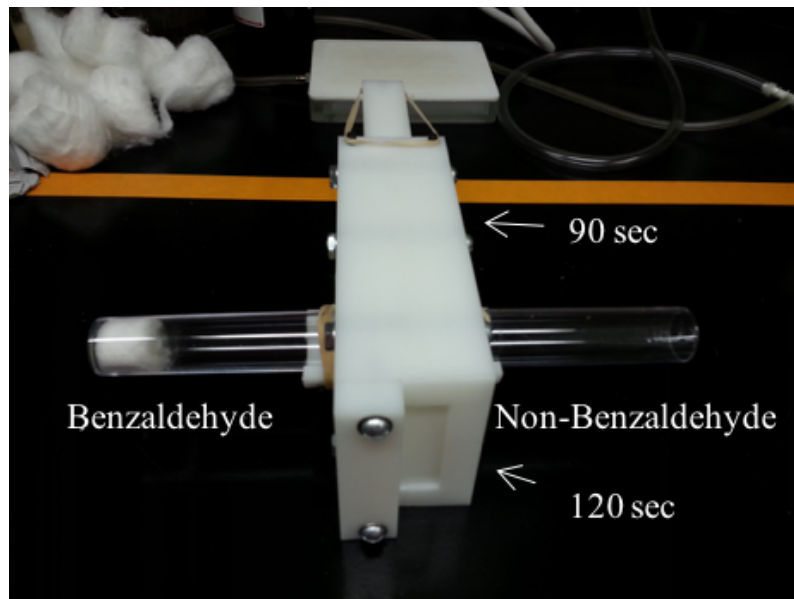

B

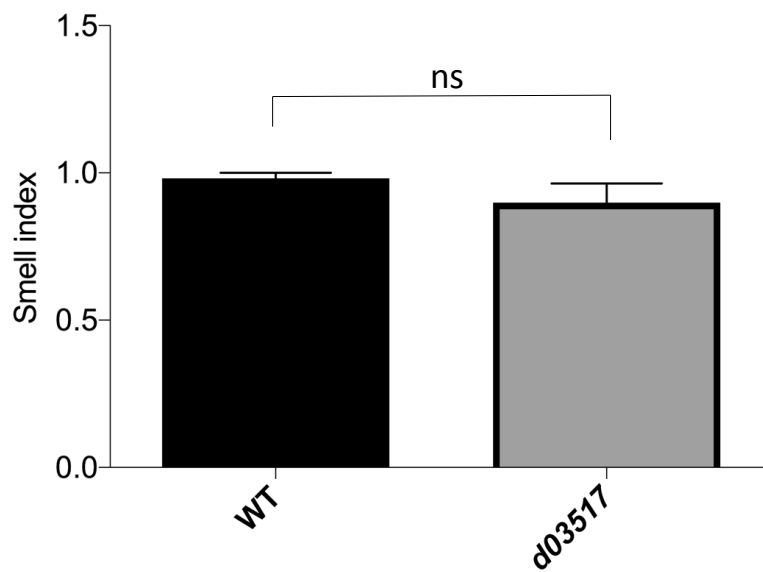

Supplementary Figure S2

Supplement: Supplementary file 4 — Figure S2. d03517 insertion did not affect olfactory avoidance response. (A) T-maze apparatus used for testing olfactory avoidance response. The apparatus consists of two separate compartments. One compartment is used for fly habituation following their introduction into the apparatus. The second compartment connects to two plastic tubes. One tube is empty, and another tube is filled with benzaldehyde. (B) Olfactory avoidance responses by wild-type and d03517 mutant flies. No significant difference between wild-type and d03517 male flies was observed (Mann-Whitney U test, “ns”, not significant, P > 0.05). Number of tests per genotype: wt, n = 12; d03517/d03517, n = 7. For each test, 10–20 flies were examined. Error bars represent SEM. Methods: Prior to the experiments, flies were deprived of food for 3-6 hours. They were then introduced into a T-maze apparatus containing two compartments (Supplementary Fig. S2A). The first compartment is for fly habituation. The second compartment connects to two plastic tubes. One tube is empty. Another tube has a cotton ball containing 1ml of benzaldehyde, a strong fruit fly repellent, at the open end. For each experiment, 10-20 flies were gently introduced into the apparatus. Flies were kept in the first compartment for 90 seconds, and then allowed to move into the second compartment for 120 seconds. Number of flies that moved into benzaldehyde-containing tube or empty tube were counted. Smell index 10.1186/s13041-018-0417-0 was then calculated as follows: Smell index= (Number of flies in empty tube-number of flies in benzaldehyde tube)/(Total number of flies). (PDF 220 kb) [file 13041_2018_417_MOESM4_ESM.pdf]

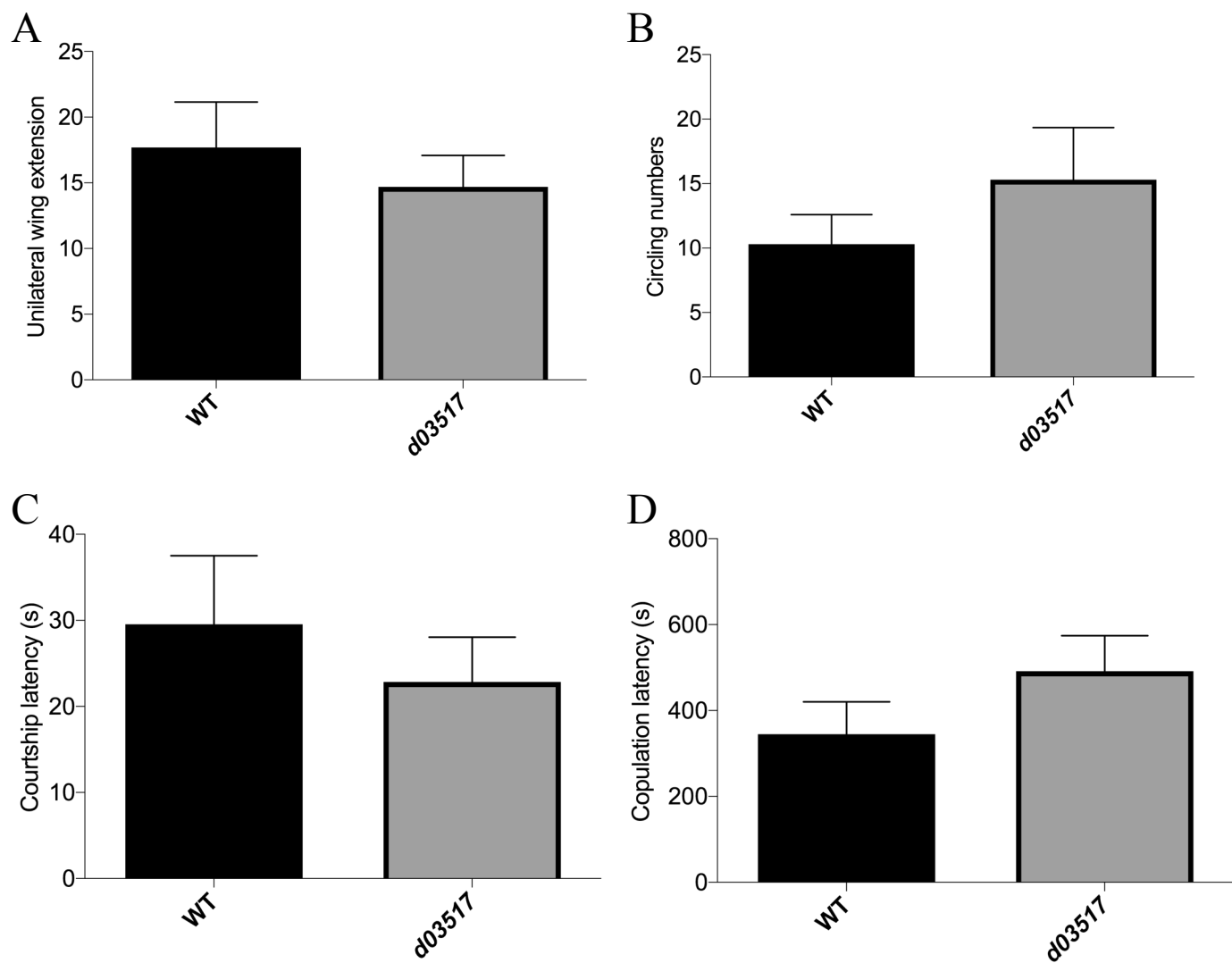

Supplementary Figure S3

Supplement: Supplementary file 6 — Figure S3. Male-female courtship behaviours for 15-min period. Wild-type and d03517 mutant male flies showed very similar male-female courtship indices (Mann-Whitney U test, P > 0.05), including one-wing extensions (A), circling frequency (B), latency to courtship (C), and latency to copulation (D). Number of flies tested: wt, n = 20; d03517/ d03517, n = 20. Error bars represent SEM. Methods: To examine male-female courtship behaviours, a CS wild-type virgin female fly was paired with a wild-type or a mutant male fly, and introduced into a rectangular chamber. Their behaviours were recorded for 15 minutes. Unilateral wing extensions and circling numbers were quantified by using the CADABRA automated analysis system. Courtship latency and copulation latency were quantified manually. (PDF 166 kb) [file 13041_2018_417_MOESM6_ESM.pdf]

A

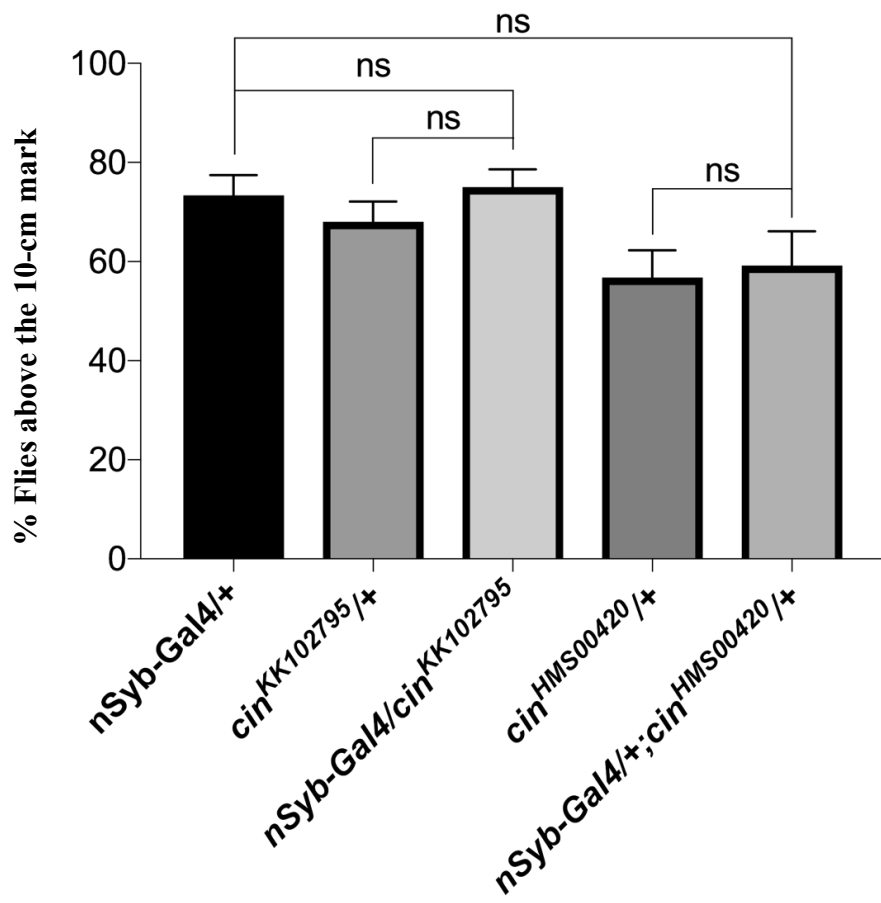

B

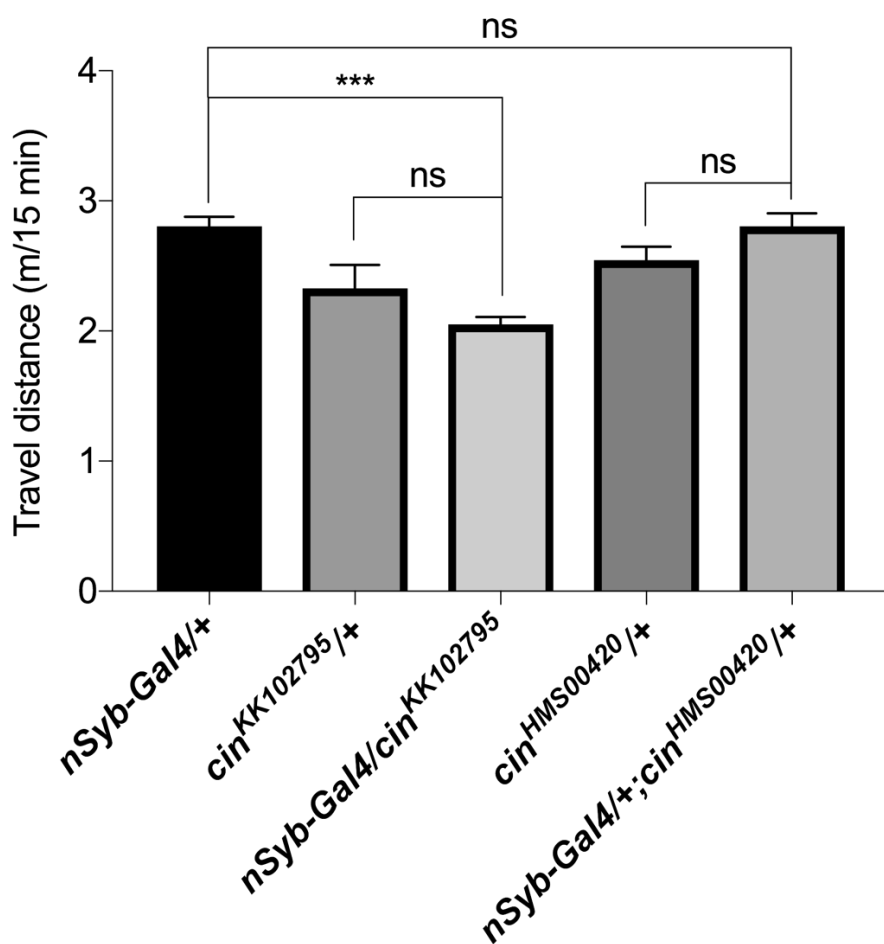

Supplementary Figure S4

Supplement: Supplementary file 8 — Figure S4. cin knockdown did not affect physical capabilities. (A) Climbing test. The percentage of flies that crossed the 10-cm mark after 15-s climbing was quantified. Number of trials: nSyb-Gal4/+, n = 27; cinKK102795/+, n = 33; nSyb-Gal4/cinKK102795, n = 36; cinHMS00420/+, n = 24; nSyb-Gal4/+;cinHMS00420/+, n = 24. No significant difference was observed between knockdown flies and control flies (Kruskal-Wallis and post hoc Mann-Whitney U tests, “ns”, not significant, P > 0.05). (B) Locomotor activity for 15-min period. Total travel distance for 15-min period was quantified. Number of individual flies tested: nSyb-Gal4/+, n = 42; cinKK102795/+, n = 48; nSyb-Gal4/cinKK102795, n = 50; cinHMS00420/+, n = 44; nSyb-Gal4/+;cinHMS00420/+, n = 46. Kruskal-Wallis and post hoc Mann-Whitney U tests, ***P < 0.001. “ns”, not significant, P > 0.05. Error bars represent SEM. (PDF 180 kb) [file 13041_2018_417_MOESM8_ESM.pdf]
